# Regulating Libra

**DOI:** 10.1093/ojls/gqaa036

**Published:** 2020-12-01

**Authors:** Dirk A Zetzsche, Ross P Buckley, Douglas W Arner

**Affiliations:** 1 Professor of Law, ADA Chair in Financial Law (Inclusive Finance), Faculty of Law, Economics and Finance, University of Luxembourg, and Director, Centre for Business and Corporate Law, Heinrich-Heine-University; 2 Australian Research Council Laureate Fellow, KPMG Law—KWM Chair of Disruptive Innovation, Scientia Professor and Member, Centre for Law, Markets and Regulation, UNSW Sydney. Email: ross.buckley@unsw.edu.au; 3 Kerry Holdings Professor in Law and Director, Asian Institute of International Financial Law, University of Hong Kong. Email: douglas.arner@hku.hk

**Keywords:** cryptocurrency, financial regulation, Facebook, digital identity, stablecoin

## Abstract

Libra is the first private cryptocurrency with the potential to change the landscape of global payment and monetary systems. Due to the scale and reach provided by its affiliation with Facebook, the question is not whether, but how, to regulate it. This article introduces the Libra project and analyses the potential responses open to regulators worldwide. We conclude that perhaps the greatest impact will come not from Libra itself, but rather from reactions to it, particularly by other BigTechs, incumbent financial institutions and governments around the world.

## 1. Introduction

Libra, the cryptocurrency project for which social media giant Facebook released the initial concept paper on 18 June 2019 (Libra 1.0), has attracted global attention. In less than two weeks, many of the world’s most influential financial regulators, including the Financial Stability Board (FSB),^1^ US Federal Reserve,^2^ Bank of England,^3^ Bundesbank^4^ and Bank of France,^5^ issued statements that their respective institutions would carefully examine Libra, and apply tough regulatory standards to it. The Group of Seven (G7) nations immediately set up a high-level forum to examine the risks of digital currencies to the financial system led by the European Central Bank,^6^ while the US House of Representatives’ Committee on Financial Services requested on 2 July 2019 that ‘Facebook and its partners immediately agree to a moratorium on any movement forward on Libra’.^7^ This was followed by a bill aimed at ‘keeping BigTech out of finance’;^8^ Facebook’s CEO Mark Zuckerberg being severely questioned by the US House of Representatives Financial Services Committee in October 2019; and demands to break up Facebook.^9^ Likewise, the Group of 20 tasked the FSB to produce a report on issues and approaches to Libra and stablecoins more generally,^10^ which was released as a consultation paper on 14 April 2020 for comment, with a final paper due to be reviewed by the G20 at its next meeting.^11^ Last but not least, Libra was the focus of a meeting at the Bank of International Settlements (BIS) that included 26 central bank governors and the BIS’s Committee on Payments and Market Infrastructure,^12^ after which the Bank of England’s Financial Policy Committee stated that, since ‘Libra has the potential to become a systemically important payment system’, ‘such a system would need to meet the highest standards of resilience and be subject to appropriate supervisory oversight’, with a framework to be adopted prior to Libra’s launch.^13^

This very high level of regulatory attention is understandable. Facebook has over 2.3 billion active monthly users globally.^14^ This scale and reach mean that the question for regulators will be how, not whether, to regulate Libra. Reflecting many of these concerns, in April 2020, on the same day as the FSB released its consultation proposal, the Libra Association released a second, substantially revised proposal (Libra 2.0).^15^

Cryptocurrencies began with Bitcoin and the thousands of subsequent Bitcoin clones.^16^ Bitcoin is a truly decentralised currency, with no central administering organisation. Its supply is very tightly constrained, so its value varies wildly. The three indicia of money are that it is a medium of exchange, a unit of account and a store of value.^17^ Bitcoin’s extreme price volatility means it can only serve as a medium of exchange in instantaneous transactions, meaning it is not money. It is also generally not considered a currency—which is a legal determination in individual jurisdictions and very few indeed have confirmed that Bitcoin is legal tender and thus currency.

From an economic perspective,^18^ Libra as initially proposed (Libra 1.0) was designed to be money. Its value would be tied to a basket of major government-issued currencies, and for each Libra issued, an equal value of such currency or highly liquid government bonds, would be placed on deposit with a reliable repository.^19^ Libra would be a stablecoin, a cryptocurrency the value of which is tied to that of fiat currency. It would not be the first stablecoin, but it would have the potential to be the first stablecoin with such breathtaking global reach and utility.

Libra is a game changer. It signals the beginning of data giants (BigTechs) entering into finance in such a fundamental way as to have the potential to usurp many of the functions of central banks, including monetary and payment systems. Years ago, Mark Zuckerberg said ‘In a lot of ways Facebook is more like a government than a traditional company’.^20^ As designed, Libra 1.0 would be his biggest step yet into the realm of the sovereign, for Libra will collect the equivalent of seigniorage—the financial benefit of issuing currency which usually accrues to a sovereign—and with Libra 1.0 would be the interest paid on the deposited cash or government bonds. While we predicted the acceleration of BigTech activities and the transformative move of BigTechs into finance,^21^ Libra is a wake-up call for all who have so far seen the data and financial economies as separate spheres, and for all who still see the issuance of currency as a unique function of the state and central banks.

This article, as the first of its kind, analyses Libra from a regulatory perspective, focusing on Libra 1.0. We start with an outline of how Libra 1.0 was intended to work and the organisation behind it in section 2, continue with Libra 1.0’s business proposition in section 3 and consider regulatory responses in section 4. In section 5, we stress the importance for cross-border cooperation in supervising Libra and analyse models that could enable cooperation. Section 6 draws conclusions about what Libra may mean for global monetary and payment systems.

## 2. The Libra Association and Consortium

### A. How Libra Works

**Figure 1:**
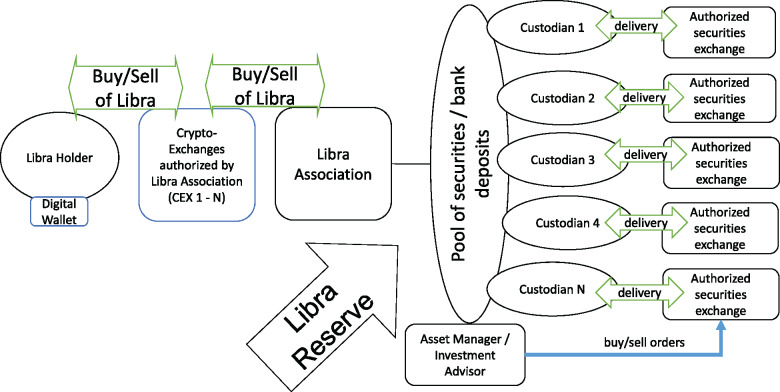
Libra 1.0.

Figure 1 depicts how we understand Libra 1.0 based on its initial White Paper released on 18 June 2019^22^ and related disclosures.^23^ We note differences between Libra 1.0 and Libra 2.0 as necessary.

Libra holders will be most likely required to have a Libra account, with a Libra custodian and/or authorised exchange. Authorised exchanges are the only institutions able to interact with the Libra Association.^24^ Once a customer swaps fiat currency into Libra, the exchange will either meet the demand by selling its own Libra stock at market price (after fees), purchasing additional Libra from other Libra holders in return for fiat currency or requesting new Libra from the Libra Association. The Libra Association is the sole issuer of Libra; only the Libra Association can ‘mint’ (ie create) new Libra or ‘burn’ (destroy) existing coins. Hence, the Libra Association functions as ‘buyer of last resort’ and ‘issuer of last resort’.^25^ Any expenses, or proceeds, respectively, of that ‘last resort’ activity will be taken from, or added to, respectively, the Libra Reserve, a pool of high-quality short-term government debt or bank deposits, designed to back all issued Libra.

At its core, Libra is a mobile money scheme, like Kenya’s M-Pesa,^26^ albeit using a cryptocurrency as the e-money and designed to run on smartphones. The main difference from M-Pesa lies in the initial scale and reach; M-Pesa needed to build a large customer base step by step, over more than a decade, responding to customer experience and complaints. Libra, however, will rely on Facebook’s distribution power to achieve scale and reach immediately.

### B. Consortium and Association

Unlike decentralised cryptocurrencies, in particular Bitcoin, Libra has a consortium underpinning its distribution and ensuring compliance with Libra’s mission as detailed in the White Paper. It will be a permissioned system and hence different from that envisioned by cryptocurrency purists. Libra is not decentralised: at the first meeting of the Libra Association in October 2019, 21 institutions from around the world formed the consortium.^27^ This was down from the 29 institutions that had signed letters of intent to join the consortium in June 2019—among them, from the payments sector, Mastercard, Mercado Pago, PayPal, Napster’s PayU, Stripe and Visa; from technology and marketplaces, Booking Holidays, eBay, Facebook/Calibra, Farfetch, Lyft, Spotify, Uber and Inc.; from telecoms, Iliad and Vodafone; from the blockchain sector, Anchorage, Bison Trails, Coinbase, Inc. and Xapo; from venture capital, Andreessen Horrowitz, Breakthrough Initiatives, Ribbit Capital, Thrive Capital and Union Square Ventures; and the non-profit organisations Creative Destruction Lab, Kiva, Mercy Corps and Women’s World Banking.^28^ Following the intense regulatory discussion,^29^ Mercado Pago, MasterCard, Visa, eBay, Stripe, Booking Holidays, Inc. and PayPal announced their unwillingness to participate in the project launch. While this means that none of the major US payment operators will initially be part of the Libra Association, these institutions reserved the right to participate in the future. Further, the Libra Association announced it had 1500 applications to join the consortium—far more than necessary to fill the 100 initial memberships, each with an entry stake of USD 10 million.

The consortium is now represented through the Libra Association, an association under Swiss law, as well as Libra Networks s.a.r.l., a limited liability company established as a Facebook subsidiary and headquartered in, and registered in the commercial register of, Geneva on 2 May 2019, with statutes dated 12 April 2019 (all Facebook’s shares were transferred to the Libra Association in October 2019^30^). Unfortunately, all Libra documentation is silent on Libra Networks s.a.r.l. However, an association cannot be licensed for financial services under Swiss law and association members cannot receive dividends, while the Libra White Paper reserves the right to pay dividends to members. Hence, we speculate that (at least) the Libra founding members will hold shares in Libra Networks.

The Libra White Paper refers to the Libra Association as a ‘non-profit organisation’.^31^ However, in the White Paper, the Association retains the right to pay fees and dividends (!) to Libra member firms. This is a highly unusual practice for a non-profit organisation,^32^ and more similar to consortia like R3, FNALITY and the original structure of Visa or, in the public sector context, international organisations like the World Bank or state-owned utilities.

### C. Governance

**Figure 2:**
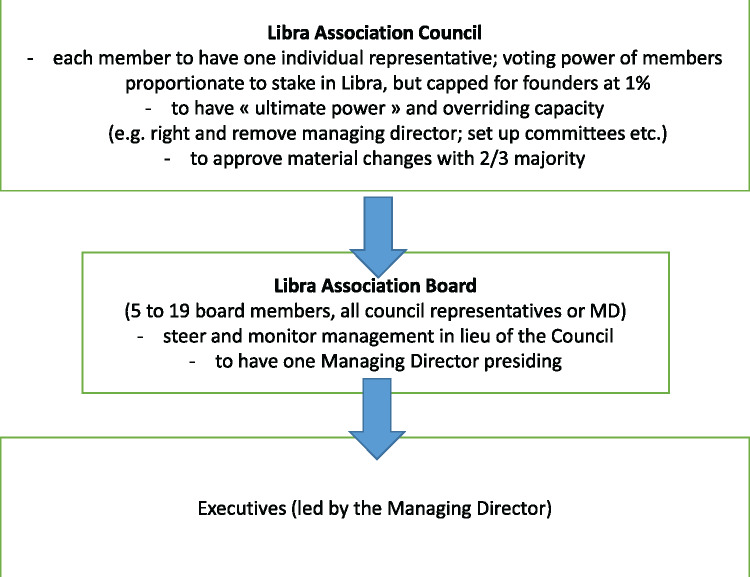
Libra’s governance.

The Libra Association serves to distance Facebook from Libra: the final decision-making authority rests with the Association, not Facebook.^33^ The expressed goal is for there to be up to 100 members of the Libra Association by the time of launch.^34^ Each will pay at least USD 10 million into Libra’s capital,^35^ in return for certain decision-making rights indicated in Figure 2.^36^

**Figure 3:**
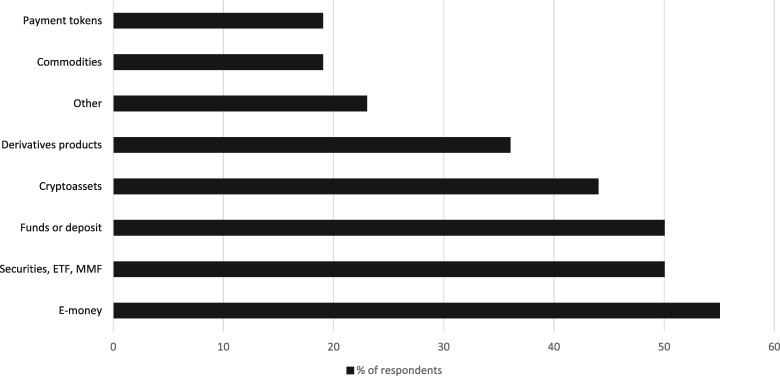
Survey among NCAs on regulatory, oversight and supervisory frameworks applicable to global stablecoins.^37^

Libra’s most striking feature, from a company law point of view, is the strong role of the council of members, particularly when compared to US companies.^38^ In line with the European concept of the limited liability company, all rights are assigned to the shareholders, and shareholders can override all board and management decisions.^39^ We speculate Libra was set up this way to appear very democratic. There may, however, be a real test of this democratic approach with the cap on voting rights of 1% for each member, as this will lead to a disproportional distribution of influence and investment. The uneven distribution of voting rights could result in free-riding of shareholders with small investments in Libra on Facebook’s and some of the other Libra founders’ large investments, resulting in *de facto* control of Facebook and the founders’ club.

At the same time, we may see opportunistic behaviour by some shareholders with small investments compared to others: the limited liability company which is the corporate form of the Libra Networks s.a.r.l. is primarily designed for business with a few—perhaps a handful—of shareholders with very large investments, and the strong rights of individual shareholders may be explained this way. Assuming that at least the 100 founding members will be, or will become, shareholders of Libra Networks s.a.r.l., the direct influence from roughly 100 shareholders is something rarely seen in this corporate form in Switzerland, or Europe for that matter. Rather, it is much more reflective of consortia structures like R3 etc. This may explain why Facebook intermediated the founding members’ influence upon Libra s.a.r.l., through transferring full ownership to the Libra Association.^40^

The Libra 1.0 White Paper characterised Facebook’s role in governance of the association as ‘equal to that of its peers’,^41^ and being fully subject to the voting cap of 1%. In particular, ‘Facebook created Calibra, a regulated subsidiary, to ensure separation between social and financial data and to build and operate services on its behalf on top of the Libra network’.^42^ During launch, Facebook was expected to take a leadership role, which would then shrink substantially over time .^43^ Given the extraordinary power of combining Facebook’s social media data with Calibra’s payments data, and given Facebook’s track record in the responsible management and use of data that included most notably the Cambridge Analytica scandal, among others,^44^ a cynic could be forgiven for doubting that Facebook will have such a tiny influence over the Association’s governance and no access to Calibra’s financial data. With the release of the Libra 2.0 White Paper, Facebook reconstituted Calibra as ‘Novi’ and clarified that it is a regulated financial company and a subsidiary of Facebook.

### D. Blockchain and Technology

Obviously Libra will use very sophisticated cryptography,^45^ but there is nothing unique about this; all sophisticated financial institutions do this to protect accounts.^46^ Libra commits to open access to the blockchain, and open infrastructure, given that ‘open access ensures low barriers to entry and innovation and encourages healthy competition that benefits consumers’.^47^

Libra will operate on a distributed ledger, but the initial processing and validating nodes will be the 21 members (later rising to up to 100 members) of the Libra Association.

Despite Libra’s establishment as a permissioned system, Facebook initially stated that full decentralisation of the blockchain for Libra 1.0 would start in five years, with the nodes being assigned influence proportionate to their overall Libra share.^48^ Given the cautious language and soft commitment in the Libra materials on this point, we believe these promises were always more announcement rhetoric than substance, designed to appeal either to ‘techies’ who love fully decentralised systems without a central governing body or to those who fear Facebook’s power. Regardless, by the time of Libra 2.0, any plans for decentralisation had been dropped.

Libra thus will be based on a permissioned blockchain. The 1.0 White Paper states that ‘as of today we do not believe that there is a proven solution that can deliver the scale, stability, and security needed to support billions of people and transactions across the globe through a permissionless network’.^49^

We agree. The governance of permissionless blockchains suffers from a number of deficiencies due to the dependency on many, for the most part anonymous and somewhat unorganised, users.^50^ One good example of these deficiencies are hard forks, ie the separation of a distributed ledger system following some users updating the system code while other users remain passive (and do not update their systems) or voluntarily decide to continue with the ‘old’ code on their servers.^51^ In the case of cryptocurrency, this hard fork would mean a split in the liquidity, the exchange processes and the reserve—a risk no prudent originator is willing to bear. By contrast, permissioned systems can agree on, and enforce, binding decision-making arrangements. However, this may come with a lower cybersecurity level and substantial potential liabilities to founders.^52^

### E. Accountability?

The prominent role of the consortium of members in the Association seems to address a major deficiency we have identified for many cryptoassets in earlier research: the lack of accountability.^53^ Of course, accountability and liability ought not be confused: Libra, as a limited liability company, erects a barrier between any liability claim and the firms and organisations which are members of the Association. In the absence of a piercing of the corporate veil (which in most major jurisdictions is very limited) and tort liability,^54^ we expect the members will be putting their reputation, but not their money (beyond their initial investment), at risk.

## 3. Libra’s Business Proposition

Libra’s mission is outlined in its June 2019 1.0 White Paper:^55^ Libra, as initially proposed, aimed to enable ‘a simple global currency and financial infrastructure that empowers billions of people’; through a ‘new decentralized blockchain, a low-volatility cryptocurrency, and a smart contract platform that together aim to create a new opportunity for responsible financial services innovation’.^56^

We have identified four elements that together characterise Libra.

### A. Financial Inclusion and Sustainability

First, Libra aims to empower billions of as yet unbanked people. While over a billion people have acquired access to financial services in the last decade,^57^ as of 2018, some 1.7 billion adults still did not have access to financial services, and many more have access but do not know how to use it effectively or wisely.^58^ As we have shown in earlier work, access to financial services is a precondition for people acting with a long-term view,^59^ and financial exclusion makes life very inefficient, with days lost in doing what should be simple tasks like paying electricity bills and with large amounts of government welfare payments disappearing in the ‘leakage’ that is common in cash and paper-based systems, particularly when combined with largely illiterate populations.

Libra, seen from this perspective, is a bold move to further the achievement of the United Nations Sustainable Development Goals (SDGs) through financial inclusion serving to assist the poor in countries around the world.

However, ‘We do not know how many people have Facebook accounts but no bank accounts’.^60^ Of the 1.7 billion people currently unbanked, over one-half of these come from just seven countries, and four of these (China, Pakistan, Indonesia and Bangladesh) have banned Facebook either permanently or temporarily at some point.^61^ It is, in fact, surprising that Libra’s promoters have not addressed or discussed this major flaw in either Libra 1.0 or 2.0. This comes in addition to the fact that many of the unbanked will not have smartphones or reliable internet access.^62^

But putting aside the reliability of some of the arguments Facebook uses to promote Libra, which financial functions could Libra actually provide?

The most important function will be cash equivalence. Libra will be a means of payment: ‘Libra will need to be accepted in many places and easy to access for those who want to use it.’^63^

As stated above, Libra is a mobile money scheme, and some commentators argue Libra will lack the cash-in/cash-out functions provided by agents—small general stores in poor countries that typically sell mobile phone airtime, mobile money, groceries and cigarettes.^64^ We expect, however, that cash-in will most likely come in government salary and welfare/transfer payments to citizens.^65^ Libra should provide poor country governments with a reliable, auditable means to get welfare payments to the intended recipients and as such could well be adopted by many governments, as well as international organisations like the UN, for instance in the context of refugees and displaced persons. Cash-out will follow as small businesses accept Libra in return for goods or services, as has happened in China with AliPay and WeChatPay.^66^ Indeed, in many poor countries, Libra is likely to generate the sort of digital financial ecosystem that mobile money advocates have long sought, particularly when combined with WhatsApp and relatively simple smartphones, both of which are becoming increasingly ubiquitous in ever more countries.

Too often today, government payments are withdrawn once transferred into mobile money accounts and thereafter the recipients transact in cash.^67^ This is inefficient and causes considerable liquidity problems for agents who function merely as cash dispensers. Libra is far more likely to underpin a digital ecosystem in which e-money is widely used, and one therefore in which fees can be much lower than is currently the case.

### B. Cost Savings

In our view, the strongest initial demand for Libra is likely to arise in poor countries where the absence of financial services—particularly lack of large-scale electronic payments systems and low-risk savings tools, often combined with lack of a sovereign digital identification system—retards development and prosperity generally.

One prominent use case should be remittances. Some of the most expensive remittance rates are from the United States to Africa, across the Gulf and South Asia, or from Australia and New Zealand to the Pacific Island nations, with costs as high as 5–10%:^68^ a Pacific Islander picking fruit in Australia today may have to spend between A$25 and A$50 to send home A$500. With Libra, that transfer should cost only a few dollars or less. Libra has the potential to replace all of these expensive current money transfer methods and, by doing so, could deliver a major global good.

In 2018, remittances exceeded aid to developing countries by a factor of about 3.5. The World Bank estimates remittances last year at about US$528 billion,^69^ compared to total official development assistance (aid) from the 30 members of the OECD’s Development Assistance Committee to such countries of some US$153 billion in 2018.^70^ Furthermore, remittances have further advantages over aid, in that remittances are more responsive than aid, ie they increase more rapidly in response to natural disasters and the like in recipient countries, and remittances inject money directly into local economies whereas much aid spending by rich countries goes to consultants from those countries who then work in capacity-building roles in the recipient countries.

Today, remittances are in effect subject to a tax, the cost of making the remittance, for which the global average was about 7 percentage points in the first quarter of 2019.^71^

These costs are legacies from times long past, when sending money around the world was difficult and expensive for financial institutions. Today, however, it is nothing more than a profit gouge by the international banks and other payments providers, and one that Libra has the potential to utterly disrupt, including for the many FinTech companies (such as Ripple and Revolut) which are already seeking to disrupt the market themselves.

So remittances should inject very considerable amounts of Libra into economies dependent on local remittance, such as those of the Philippines, Nepal, Samoa, Tonga and Bangladesh. It would be surprising, given these injections of liquidity, if local merchants in these countries do not quickly begin accepting Libra in payment for goods and services. This will likely be particularly so in those countries where Facebook and/or WhatsApp use are already very common, such as the Philippines, Bangladesh and India.^72^

Demand in developed countries is less easy to predict, but presumably this is why firms such as Uber, Lyft, Spotify, Amazon and eBay were invited to join the Association. Uber alone currently pays over US$800 million annually for credit card merchant fees.^73^ We would thus expect generous discounts from Uber, Lyft, Amazon and others for paying in Libra. Such tech companies often engage in below-cost pricing for long periods, seeking market dominance and long-term, rather than short-term, profitability. Discounts on payments in Libra would fit into this pattern of behaviour and give rich country consumers a reason to adopt the currency.

### C. ‘Stable Coin’

Initially customers will buy Libra by paying fiat currency. The Libra Association will then put this currency on deposit with a repository or use it to buy highly liquid government bonds and entrust them to the repository. Libra will function as a so-called stablecoin tied to major government fiat currencies. Libra aims to ensure people’s ‘confidence that they can use Libra and that its value will remain relatively stable over time’.^74^

In its initial form, it is apparent from this that, besides cash equivalence, Libra 1.0 could also provide a currency hedge. Many currencies of developing countries are impossible to hedge, for lack of market liquidity: no one wants to hold them as a long position which is necessary for the other side to go short. This has driven up hedging costs for many poorer countries like Cambodia, Samoa and Guatemala to above 10%.

Given its potential liquidity and the ability to exchange both major and minor currencies for Libra at the net asset value of the basket of major currencies, Libra 1.0 offered dramatic potential to provide both a low-cost tool for hedging currency risk and also for directly reducing exchange costs for developing country currencies (which are generally traded against a major currency, usually the US dollar, in the centre of any developing country cross-currency exchange, thereby increasing costs as well as risks).

The potential for use in hedging depends on the currency the exchanges or the Libra Reserve accepts in return for Libra. Given the enormous scale, and potential worldwide exposures, hedging could become less expensive if the Libra Reserve engages in (very) skilled risk management. The hedging ability, of course, depends on the composition of the Reserve’s basket. Initially, there was very limited reliable detail regarding the composition of the reserve: whether it would be along the lines of special drawing rights (in particular, the IMF Special Drawing Rights (SDRs), comprising the US dollar, euro, yen, pound sterling and RMB) or trade or otherwise weighted (to incorporate a wide range of currencies, potentially even a universal index), or none of the above.^75^ In September 2019, Facebook clarified in responses to questions to the US Congress regarding the potential role of the Chinese yuan in the basket, confirming that the basket would comprise initially the US dollar, euro, yen, pound sterling and Singapore dollar.^76^ This composition would in fact be similar to the SDR, albeit with the Singapore dollar included instead of the Chinese yuan.

We note, however, that Libra 1.0 was not to be a panacea to all difficulties residents of developing countries face with regard to their local, in many cases scarcely traded (‘illiquid’), currency, characterised by supply in that currency constantly exceeding the demand in currency markets.^77^ From what we can see in the Libra 1.0 White Paper, in return for minting Libra, the Libra Reserve would take in stable, liquid currency only. Illiquid currency would then remain with the Libra exchanges. However, since supply in those currencies typically exceeds demand, the exchanges would not want to have such currency on their balance sheet; thus, we would have expected the exchanges to charge clients for the potential losses from accepting the illiquid currency in the first place, either directly as fees or indirectly via the exchange rate. These costs could be significant: currency exchanges accepting illiquid currency currently charge two-digit percentage points costs to clients; and Libra exchanges are likely to do likewise.

Libra 2.0, however, will constitute a series of individual major-currency stablecoins, exchangeable into individual major fiat currencies and backed by individual major fiat currencies, but with the possibility of acquiring an asset comprising a mixed basket of these coins. Thus, Libra 2.0 faces some of the same challenges as other major fiat currencies: at what cost (spread) will exchanges transact domestic currencies into individual Libra single currency stablecoins or into the Libra mixed currency stablecoin?

### D. Disruptive Potential: Why Banks Should Be Afraid

The cost savings Libra offers come at someone else’s expense, and that someone will typically be incumbent financial institutions and new FinTech entrants. The transformative nature of Libra lies in Facebook’s reach. It is expected that Novi, Facebook’s new digital wallet provider for Libra, will be available through Facebook Messenger, WhatsApp and Instagram, the Facebook applications through which it reaches billions of customers.^78^

Libra’s potential to disrupt incumbent banking in the developed world is massive. Libra would propel Facebook to the top of the queue of BigTechs seeking to outcompete the banks. This would happen for two reasons.

First, Facebook has better access to more data than incumbent banks and payments providers. Historically, incumbent banks all over the world have had the best data on customers and have therefore been best placed to price credit and insurance.^79^ Facebook’s data advantages change that. The cozy old world in which a banking licence was an exorbitant privilege is coming to an end, and fast. Data-driven disruption is far more likely than people think. In China, Ant Financial, the financial services subsidiary of Alibaba, uses its vast store of data to be a leading consumer lender and financial services supplier. In America, two of the leading small business lenders are Amazon and Square, a payments app. Ant, Amazon and Square have better data than the banks, and they have a real-time feed on business income as it is paid by customers, so of course they are displacing incumbent banks as lenders. The combination of Facebook’s social media data with the payments data of Libra will be transformatively powerful.

Secondly, the Libra ecosystem will create self-reinforcing network effects and economies of scope and scale: the more people use Libra, the more applications for Libra will be written, attracting even more users to Libra. A massive client base such as Facebook’s is an excellent starting point from which to create enormous platform effects.

Libra may not win the cryptocurrency race, but it is a game changer. Radically new strategic thinking will be required of incumbent financial services firms for them to respond. Bankers will need to learn to deal with data. Data companies see the world differently and in ways that, in finance, are far more powerful and profitable than the perspectives of traditional banks. The question is whether our banks’ leaders will be up to the challenge. In datafied finance, the lender with the best data and data analytics wins.

Unless the regulators deliberately seek to thwart the growth of Libra (see section 4 below), the lender with the best data may well be Facebook, or some other BigTech that follows Facebook’s lead and offers its own cryptocurrency in the context of FSB-agreed regulatory and supervisory approaches: Amazon Coin or Google Coin anyone?

In fact, we suggest that one of the greatest impacts of Libra may well be that it will prove to be the first of a range of similar proposals, from a range of both private and public organisations. We suspect that these will include stablecoin offerings by other BigTechs, as well as incumbent financial institutions and FinTechs, governments and possibly international organisations. Many governments have done extensive work preparing to issue a central bank digital currency,^80^ yet no credible government^81^ has yet issued a central bank digital currency with retail access,^82^ as doing so would mean reworking the financial system in fundamental ways, the consequences of which would be very difficult to predict.^83^ However, this is becoming increasingly likely, with China and Sweden leading contenders, with serious discussions ongoing in the Eurozone and the United States as well. It may be that the best sovereign response to Libra or other private global stablecoins will be for governments to counter with their own digital currency.^84^

## 4. Regulatory Concerns

A plethora of regulatory concerns accompany the Libra project, and regulators around the world have already made clear they will require high regulatory standards, given Libra’s scale and reach. We expect regulators to act in the three primary regulatory paradigms when regulating Libra. These include *consumer protection* (also referred to as investor, customer, client and/or depositor protection), the protection of *financial stability and market functions* (including systemic risk) and *market integrity* (particularly around potential for criminal use). These will be joined by macroeconomic, political and stakeholder concerns, and as Libra can substitute for fiat currency, political, monetary and financial stability concerns will be key in this regard. The April 2020 FSB proposals^85^ very clearly reflect exactly this framework and set of concerns.

### A. Licensing

As a starting point, Facebook/Libra will certainly be required to obtain a range of licences across a number of jurisdictions and to comply with existing anti-money laundering (AML) and countering the financing of terrorism (CFT) regulations.^86^ Some of the potential licence requirements are considered here, with more to follow, once more details about Libra are released. We delineate between two types of licences: those that relate to Libra’s issuing services and those that relate to the cryptoasset itself.

#### (i) Libra’s services

Licences will likely be required for one or several of the services of Libra.

First, Libra will need licences to provide payment services in a range of jurisdictions, as this is a regulated activity around the world, particularly when there are public interest concerns around consumer protection, financial stability and market integrity, as are potentially evident in this case. Libra could submit itself to the stringent requirements of ‘systemically important payment systems’ harmonised through the BIS/International Organization of Securities Commissions (IOSCO) accord (which comes with the main advantage of exemptions from many national licensing requirements regarding Libra’s core payment facilities, in return for oversight by a lead supervising authority,^87^ with all other competent authorities joining in a supervisors’ college),^88^ while Libra’s agents (the exchanges, risk managers etc) could apply for payment or other service providers’ licences in many different countries, including as a money transmitter in the United States and under the Payment Service Directive (PSD)^89^ in the EU. Payment service providers offer receiving entities (such as retail, commercial or public clients) services for accepting bank-based and online payments. Many jurisdictions have payment service provider schemes which could, and in all likelihood would, be applied.

While it is uncertain whether the Libra Reserve meets the requirements set for payment systems,^90^ the Libra Association has applied for, and the Swiss Financial Market Supervisory Authority (FINMA) have confirmed in principle (subject to conditions and additional requirements), that ‘stablecoins’ qualify for a payment systems licence.^91^ Interestingly, while FINMA committed to apply the BIS/IOSCO framework for payment systems, the Swiss law does not have a set of rules equivalent to the PSD governing service *vis-à-vis* EU *retail* clients, nor does a Swiss payment system licence qualify for such services. So Libra, or its authorised exchanges, will need additional PSD licences in the EU.

It is the advantage of the EU’s PSD licence that it comes with a European passport, which is the right to provide payment services across borders, ie one PSD licence works for 30/31^92^ EU and EEA countries. We expect Novito to acquire an EU licence—in addition to a US licence—as necessary. In return, the provider needs, most notably, to segregate and safeguard all funds which have been received from the payment service users or through another payment service provider for the execution of payment transactions.^93^

Secondly, some jurisdictions require licences for e-money providers. In particular, under EU financial legislation, if the provider does not qualify for payment services, an e-money licence subject to the E-Money Directive^94^ may be the measure of choice.^95^ E-money is often defined as a digital alternative to cash, allowing users to make cashless payments over the internet, the alternatives being a card or a phone. EU rules on e-money aim to facilitate the emergence of new, innovative and secure e-money services, and encourage effective competition between all market participants. Similar to the PSD licence, the EU e-money licence comes with a European passport. A range of other jurisdictions around the world have similar requirements.

Thirdly, in many jurisdictions, payment services are still limited to banks and in the absence of alternative payment and/or e-money licences schemes, Libra may have to acquire banking licences in some jurisdictions.^96^

Other types of licences depend on regulators’ interpretation of Libra’s services. For instance, such licences could follow from the qualification as an investment fund/collective investment scheme. The main features of an investment fund/collective scheme is that an asset pool is managed by a third-party expert and the contributors in the pool share the proceeds of the pool.^97^ If Libra holders participate—at least partially—in the proceeds of the Libra Reserve, regulators could characterise Libra’s set-up as a money market fund, and demand a licence for the fund and the fund’s management (under the UCITS Directive^98^ in the EU, or the Investment Company Act and the Investment Advisers’ Act in the United States^99^). Qualification as an investment fund would be supported by the fact that Libra users’ confidence is supported by a reserve pool of high-quality investments, usually government bonds and bank deposits,^100^ and all Libra sold for fiat currency will entitle the holder thereof to a share in the pool.^101^ Compare this with money market funds, which tend to invest in government debt and short-term deposits only,^102^ similar to Libra’s Reserve, where holders are exposed to the returns of the asset pool: money market funds with cash-equivalent functions, ensured through a fixed net asset value, were very successful in the United States, until they experienced a crisis when the nominal value of one unit deviated from 1 USD (‘breaking the buck’),^103^ resulting in global attention to the issue from regulators.^104^ The risk of being qualified as a money market fund may help explain why Libra 2.0 refrains from allocating profits generated through the Reserve to Libra holders, contrary to the original design in Libra 1.0.

As Libra’s services expand, it will have to acquire additional licences around the world. For example, if the Libra organisation decides to accept deposits on behalf of clients, it will need a licence as a bank or credit institution, potentially in every jurisdiction in which it seeks to provide such services.

Finally, Libra will require licences for its custody and safekeeping systems, which underlie the link between the basket of fiat currencies and Libra. If not for other reasons, these services alone could drive Libra’s rise to the level of systemically important payment and settlement infrastructure in some jurisdictions.^105^

#### (ii) Coin characteristics

We have laid out in previous work that cryptoassets can be characterised as financial products of many different kinds.^106^ This is not the place to repeat the discussion. Suffice to say that any cryptoasset could potentially be understood as money, currency, a payment instrument or system, a security, a commodity and/or a financial derivative, or several or none of the above.^107^

If not qualified as a collective investment scheme (see section 4A(i)), Libra could be qualified as comprising a commodity or a financial derivative, with each Libra coin representing rights in a basket of cash on deposit and highly liquid government bonds. The arrangement could be structured as flow-through (analogising Libra to collective investment schemes or structured deposits) or as a securitisation (rendering Libra a structured security).

The characterisation as commodity, investment fund/collective investment scheme and/or financial derivative will also determine the licensing conditions for service providers such as the authorised exchanges that trade in Libra and custodians that offer Libra accounts. Certainly the major US and EU regulators, joining forces in the G7 Working Group on Stablecoins,^108^ have already indicated the necessity of discussions with Facebook about determining appropriate regulatory treatment (as well as prompting a discussion on how to supervise ‘global stablecoins’ in general, led by the FSB). One crucial point of these discussions are the exchanges. In addition to, or alternative to, the payments frameworks, we would expect it to be necessary for the exchanges to obtain a licence as a commodity dealer in the United States and/or in the EU to be licensed as an investment firm under the Markets in Financial Instruments Directive (MiFID^109^) for reception and transmission of orders, dealing on own account and execution of orders in relation to, or placing of, financial instruments, respectively.^110^

These regulations will be sorely needed as the cryptoexchanges have proven to be the point of vulnerability for cryptoasset investors. Nearly all the major losses in cryptoassets have come through attacks on the exchanges^111^ or their operational deficiencies, or from conflicts of interest arising from their acting simultaneously as exchanges and custodians.^112^ Fundamental to all these regulatory schemes are systems of custody of assets and segregation of accounts, as well as a range of requirements relating to market integrity, such as AML/CFT customer due diligence (CDD).^113^ These aspects are grossly underdeveloped in the proposals at this stage.

Depending on the Libra Association’s scope of activities, Libra could also qualify as an issuer of a derivative and a trader in those assets which would subject the Association to the need to obtain a licence as a broker-dealer or commodity dealer (United States) or to be licensed under the MiFID as an investment firm (EU).^114^ Along with these regulatory requirements will come, for instance, custody, segregation and compliance requirements.^115^

#### (iii) Managing the Reserve pool

It has not yet been disclosed how the Reserve pool will be structured legally. There are two main alternatives. On the one hand, the Libra Association could become the owner of the Libra Reserve and manage its own assets. However, this would subject the pool to all claims of creditors of the Libra Association, including, for instance, fines for antitrust, data protection and foreign trade violations, which could reach an enormous scale (especially in the EU^116^), and Libra’s own tax liabilities. If structured in this way, Libra’s net asset value could be potentially severely impaired, and the current White Paper disclosure would be misleading, so this structure is highly unlikely.

Thus, we expect that the Reserve Pool will be managed on behalf of the Libra holders as beneficiaries, through a special purpose vehicle earmarked for this purpose, a collective investment scheme or a trust arrangement. In this case, the Libra Association must obtain an investment adviser licence or an asset or fund manager licence, or employ an external advisor/manager for the purpose.

With regard to the custody and safekeeping of Libra’s reserve, what we have stated above in section 4A(i) applies *mutatis mutandis*: custody and safekeeping is required of licensed custodian banks and/or investment firms.

### B. Risk Management

Libra is a stablecoin, but ‘stablecoin’ is something of a misnomer as its stability will rest on a number of operational and financial preconditions that regulators will want to ensure through regulation. Libra 1.0 in particular raised a range of challenges, most of which have been scaled back in Libra 2.0.

#### (i) Operational risk

First, operating the Libra Reserve professionally and preventing Libra holders from generating operational risk is key. For instance, the distribution of the Libra reserve fiat currency should happen as instructed by the asset manager in charge, with appropriate protections in place to ensure no one can steal from the portfolio backing Libra, or err when transferring fiat currency received from Libra users to the reserve account. The Libra Reserve’s net asset value will need to be calculated several times a day, accurately and with an eye to preventing market abuse and insider trading, in addition to fraud, theft and a significant degree of cyberrisk.^117^ All of these concerns will warrant extensive operational safeguards and justify licensing requirements as asset managers, investment advisors or investment fund managers.

#### (ii) Financial risk

Secondly, Libra promises stability.^118^ To achieve this, skilled asset managers must determine the portfolio composition of the Reserve basket, and rebalance the portfolio on a daily basis. In Libra 1.0, this would have had to be done on a multi-currency basket structure, across multiple currencies and time zones, and potentially multiple trading venues across the globe. Without this, the multi-currency reserve concept is unlikely to work, resulting in price differences between the notional and reserve values, prompting the risk of a ‘run on Libra’.^119^

Regardless of how stability is weighted in the asset manager’s composition, a stablecoin is never really stable from a Libra 1.0 holder’s perspective. Even where the basket is well diversified, the value of the basket will fluctuate in line with overall (global) market swings. Only multinational firms whose exposures are similar to the Libra 1.0 Reserve’s currency basket (if any) would understand Libra 1.0 as ‘stable’; for the rest (ie all retail holders), Libra’s value is likely to fluctuate.

Reflecting this, Libra 2.0 supplemented the multi-currency structure with a series of single currency fiat-backed stablecoins, and by expressly stating that the multi-currency stablecoin will be created as a synthesis of a basket of the single currency ones, similar to a synthetically structured currency index fund.^120^

Nonetheless, the issue remains, with respect to anyone not from the jurisdiction of the individual major currency stablecoin: how this fluctuation correlates with the Libra holder’s home currency will depend on the holder’s home country. From the perspective of the Venezuelan bolivar, Libra may be relatively stable, while from the perspective of the US dollar, euro or Swiss franc, the fluctuation prompted by mixing additional currencies in the (albeit synthetic) basket may be experienced as less stable than the holder’s home currency.

#### (iii) Systemic risk?

The third systemic risk is a concern under both the too-big-too-fail (TBTF) and too-connected-to-fail (TCTF) paradigms.

As to TBTF, we can only guess how many of Facebook’s clients, and how many of the currently unbanked, will buy and use Libra; and we have no reliable data on the funds a single client will swap into Libra. Estimates in the press suggest an overall Libra market volume equivalent to US$100–500 billion.^121^ However, these are pure guesses, and it could of course be much more if Libra becomes the coin of fashion among Facebook and WhatsApp users around the world.

As to TCTF, the Libra Association will be at the heart of a new financial ecosystem on which millions of Libra holders and thousands of merchants and service providers will depend. This interconnectivity is both financial and technical in nature.^122^

Hence, Libra raises some old, but also entirely new, forms of systemic risk. We would expect, and in fact encourage, within a very short time, that Libra, in its capacity as a crucial payment system provider, or bank respectively, would be brought within the global framework^123^ addressing globally systemically important financial institutions (G-SIFIs^124^) and/or systemically important financial infrastructure, including a systemic risk surcharge, similar to that charged to existing G-SIFIs.

Libra is perhaps the ultimate example of something that is highly likely to move from ‘too small to care’ to ‘too big to fail’ very quickly.^125^ The potential for Libra to become systemically significant within a few months of launch in some markets prompts us to issue, again, the warning we have delivered with regard to data-driven finance in previous work:^126^ Libra will not only be a currency in some countries, but the Libra ecosystem will likely become an important payments and capital market infrastructure. In the big data age, financial regulators should also consider market structure as central to their function, rather than the exclusive domain of competition/antitrust regulators.^127^

Thus, it is not surprising that the initial outcry from regulators around the world triggered a radically more collaborative and cooperative approach between the Libra Association, Facebook and global regulators in the context of Libra 2.0.

### C. Capital Requirements

We need to distinguish between two aspects of capital requirements. First, there is the capital required to back up the stablecoin. If the capital pool is segregated, as we recommend, no additional capital must be put up for the liabilities resulting from the contractual obligations *vis-à-vis* Libra holders.

However, the Libra Association will need to provide for capital to ensure operational consistency. Given the enormous amount of assets held in the Libra pool and the additional complexity associated with synthetic pooling, we would recommend a capital requirement analogous to that for investment firms and fund managers for operational risk. Since none of these licences cover all countries, and group approaches for these firms have not been established across the globe, licensing requirements across countries would be cumulative.

### D. Identity and AML

In all countries, regulators will require Facebook to conduct AML/CFT/CDD checks on Libra users.^128^ The Libra plan includes a digital identity to meet these requirements.^129^ Once Facebook achieves this regulatory compliance, as it will with technology and its financial resources, it will have overcome a major barrier to the offering of financial services, and will start offering more of them. From our standpoint, this aspect of Libra, a global digital identity solution, may well prove even more powerfully transformative than the cryptocurrency itself.

As we have examined in previous work,^130^ digital identification is crucial to financial inclusion and to achieving the UN SDGs more broadly. This is stressed in the Libra White Paper in Libra’s commitment to digital identity.^131^ The Libra documents remain silent, however, about the real challenge, which is how to achieve a digital identity for many of the 1.7 billion unbanked people who hold neither a passport nor other identity document.

Libra offers all the opportunities for a scheme we have proposed in our earlier work to employ not only business identity (offered by Libra, among others), but also individual identification (eg through biometric means),^132^ in a way currently not politically imaginable in many countries, so as to recreate the official centralised identity of the unbanked. Regulators will want to cooperate with Libra and others to make use of this unique opportunity; and if they do not move swiftly, the Libra ID may well become the *de facto* new identity for both AML/CTF/CDD and other purposes, without their involvement, as has happened to an extent in China with the digital identities created and conferred by AliPay and WeChatPay. Given Facebook’s history with customer data,^133^ however, this may raise far bigger concerns about privacy and data protection than the Libra cryptocurrency.

### E. Monetary Policy

If Libra succeeds in developing countries as we expect it will, and as have M-Pesa in East Africa and AliPay and WeChatPay in China, it will pose fundamental challenges to governments, as it will in many cases shift substantial control of monetary policy from governments to the Libra Association. Libra will insert a private company between national central banks and the citizens they seek to serve. Furthermore, once well established, Libra’s global nature will mean capital controls will no longer be a policy measure available to governments to prevent capital flight in times of severe economic uncertainty (as Malaysia did in 1998 or as China has done over the last several decades).^134^ Its impact on the monetary supply and consequently monetary policy of emerging markets nations is potentially very systemically destabilising. Major current policy tools for poor country governments may be denied them.

The world’s major financial regulators needed time to assess and regulate Libra, and, as we expected, they created it by slowing Libra’s growth in many ways (most notably, by asking for detailed information multiple times over) to preserve the stability of the global financial system.

Despite these very sizeable risks, some potential outcomes are potentially positive for users. Among the largest users of Bitcoin are those living in poor countries with weak institutional environments, with Zimbabwe and Venezuela paradigmatic examples where Bitcoin provides an alternative to problematic national currency and monetary systems. It is thus possible that Libra could in fact force governments to do better in managing their own economies and currencies, in the way the gold standard did prior to World War I. There is certainly the possibility of the emergence of a better alternative in many cases through Libra. It does, however, raise particular concerns about the potential power of a private consortium underpinning global monetary arrangements, highlighting the potential value in an international regulatory approach.

One other feature of Libra puts its acceptance by developing countries at risk. We can reasonably assume that most custodians holding the Libra Reserve will be located outside developing countries. Thus, countries with a large Libra acceptance will suffer from a fiat currency outflow.

As unilateralism seemingly is increasingly becoming a newly accepted policy path, we may see the reserves from certain countries being held hostage in the name of foreign sovereign, or international commercial, policy: authorities could order exchanges to avoid transactions with Libra holders of any given country, in order to bring pressure to bear on that country’s government. In order to retain sovereignty, regulators could require local custody of Libra Reserve funds in the home currency equivalent to the amount of Libra circulating in any given country, thereby putting at risk the business propositions of independence and stability independent of local circumstances.

### F. Data Protection

Facebook can leverage its client base to Libra if it can use Facebook clients’ data at least for the initial contact. The Libra 1.0 White Paper states that ‘Facebook created Calibra, a regulated subsidiary, to ensure separation between social and financial data and to build and operate services on its behalf on top of the Libra network’.^135^

In short, Calibra—now Novi—is the entity charged with turning Libra into a business for Facebook and—other than the name—this reality does not change appreciably with Libra 2.0.

Transferring client data to Libra, or Novi, Facebook’s digital wallet provider for Libra storage,^136^ would require the clients’ consent at least under EU, UK and Australian data protection law, given that Facebook clients have consented to use of their data for social media, rather than financial services, purposes. We have not found any clear language that ensures permanent data separation between Facebook and Novi in Libra’s materials, and concerns that both data pools may be mixed with or without users’ consent are valid, given Facebook’s history of continual data protection violations in the last decade.^137^ If both data pools were to be merged—as seems likely in the context of Libra 2.0, Facebook would have unprecedented insight into and control over their users’ social and financial existence.

### G. Tax

Finally, tax is a valid concern. For instance, regulators need to determine whether VAT is charged on transactions, and whether profits on the sale of Libra attract capital gains tax. Further attention is also warranted with regard to the Libra Reserve’s proceeds, how they are generated and if, and how, they are going to be taxed.

If a regulator in a given country wants to slow the take-up of Libra severely, declaring that profits on the holding of Libra would be subject to capital gains tax may prove to be the policy option of choice. In countries in which local currencies tend over time to devalue, such a resolution of the taxation authority would require holders of Libra to pay capital gains tax each time they use their Libra to make a purchase. This could render the currency functionally unusable provided the country has the means to enforce such a measure.

### H. Disclosures

All of the issues we have outlined must be the subject of adequate analysis and disclosure. Similar to other initial coin offering white papers we have analysed,^138^ there is currently very little financial detail in the Libra documents, and too little information to allow consumers to assess whether purchasing Libra is a good idea.

To name but one example of incomplete disclosure, managing billions, if not trillions, of dollars, albeit on a low-risk stability basis and within a low interest environment, will yield considerable returns, and these returns are potentially very large when compared to the initial investments made by Libra founding members, given that the proceeds depend on the fiat currency contributed by Libra holders rather than the Libra members’ initial investment. The Libra 1.0 White Paper is thin in this regard. For instance, while Libra is dubbed a non-profit organisation, its members are entitled to a dividend,^139^ and some profits from the Libra Reserve will be used to fund expansion of the Libra project, while Libra holders will never get a dividend.^140^ Reasonable Libra holders will expect far more details on the conditions on which proceeds from the Libra Reserve will be reinvested in the network, retained as cash equivalent or paid out to Libra founding members.

Reflecting these concerns, Libra 2.0 makes clear that the seigniorage will not devolve to the Libra Association but rather will remain with the fiat issuing central banks.

## 5. Cross-Border Supervision

Libra is a global project. Given the current state of international financial regulation, the best we could hope for Libra is a group of regulated and capitalised subsidiaries under a main holding entity, with cross-border cooperation and coordination through supervisory colleges of involved supervisors, chaired by the main supervisor of Libra’s home jurisdiction/holding entity.

Libra will fall under many different national and regional licensing and supervision regimes. Most regulators will respond to Libra and impose additional conditions reflecting the national perspective.^141^ If it all happens, overcoming this diversity of views will take time, but more likely is a mixed and potentially fragmented regulatory framework which will limit some of Libra’s advantages. More importantly, a highly fragmented regulatory landscape will lead to inefficient regulation. This is the background of the recent FSB approach to ensure consistent regulatory treatment of ‘global stablecoins’ globally. So how to ensure effective cross-border supervision?

The obvious answer for financial lawyers is substituted compliance, or in European law terms: equivalence.^142^ Once Libra is licensed in one country, other countries recognise its supervision in the home country and reduce their own requirements, for instance on risk capital, risk management and IT infrastructure, under the condition that the financial legislation and supervision in the home country has substantially the same effects as the legislation and supervision in the host country, and the home country regulator commits to ensure protection of host country clients to the same extent as home country clients. We foresee a number of issues when applying substituted compliance to Libra.

First, the most important regulators all seem to want to have their say on Libra, given that Libra clients in their own country will be subject to the risks Libra creates. As is well evidenced by the US/China trade conflict, multilateralism is in crisis, with very important economies preferring a unilateral approach over trusting other countries. This is particularly true for monetary policy where the present US government is dissatisfied with the currency ratio of the US dollar versus the yuan and the euro, and criticising the respective central banks for apparent currency manipulation to the detriment of the United States.^143^ In turn, US regulators ask for an important role in Libra’s regulation and supervision. This has led the Libra Association to assure US regulators that it will not proceed with its global business until US concerns have been addressed.^144^ If Libra grants the same privilege to other major regulators (such as the EU, UK and Japan), the slowest will control the pace at which Libra proceeds.

Secondly, outside of the European Union, where equivalence is established as a principle in most types of financial services (yet very few countries have been approved under the equivalence tests, for legal and political concerns, and any review is likely to be stricter after the expiry of the transition period following the UK’s departure from the EU), the multilateral supervision framework is not strongly developed. In particular, outside of derivatives regulation, substituted compliance has found few friends in the United States, whose regulators demand, for obvious reasons, a say in regulating Libra. In banking, where cross-border cooperation has a long history dating back to the late 1960s, it is clear that outside of passporting in the EU (and some countries privileged under the European Commission’s equivalence assessment), home country supervision has largely fallen out of favour, with even the US rarely now arguing for cross-border branch-based regulation and the EU having moved to centralised banking supervision under the Single Supervisory Mechanism.^145^

Thirdly, the scope of substituted compliance is patchy at best, and expanding the scope will face major difficulties, including creating trust in the respective foreign regulators. This will be particularly important in the case of Libra, where the structure will reside in Switzerland but will operate around the globe. Even where central bankers are willing to adopt a multilateral framework (such as the BIS/IOSCO approach to payment systems or the FSB framework of supervisory colleges applied to G-SIFIs) to ensure global supervision, it is far from certain that any new cross-border joint supervision scheme will find support at home, while Facebook’s high profile and poor track record ensure that this will be a hot political issue in many countries.

## 6. Conclusion

Given Libra’s potential scale once Facebook links its massive client base via Messenger, WhatsApp and Instagram to Libra via Novi, worldwide monetary and financial regulators have no choice but to regulate Libra and are moving to do so on a global basis. This article has outlined a number of regulatory concerns. In fact, even without Libra the cryptocurrency, this is already the case for Facebook Pay (which operates across Facebook, Messenger, WhatsApp and Instagram), launched in the United States in October 2019,^146^ and with WhatsApp Pay awaiting regulatory approval in India since December 2018 to extend to over 300 million Indian WhatsApp users.^147^

The key problem in regulating Libra will likely be that cross-border supervisory cooperation and co-supervision schemes are patchy, and little tested for financial services, beyond banking and derivatives. Without a multilateral approach to, at least, Libra’s Reserve, Libra 1.0 was likely to fail. Establishing new multilateral systems, however, would take much longer than Libra would need to get operational and are unlikely given current geopolitical trends. Not surprisingly, this element of Libra 1.0 has been replaced in Libra 2.0 by a much simpler single currency stablecoin structure.

Libra challenges the major international regulators to move with unprecedented speed and cooperation, and we see that a genuine attempt to meet this challenge will be accompanied by some regulatory roadblocks to slow Libra’s development and buy the regulatory community more time within which to respond comprehensively, as evidenced by the FSB’s April 2020 proposals.

Looking forward, we suggest the greatest impact of Libra may be to trigger a range of similar proposals from other BigTechs and incumbent financial institutions, some of which may be better than Libra. We also suggest it is highly likely Libra will lead one or more of the major currency central banks/governments to move forward with a sovereign digital currency—with China likely to be the first.^148^ Furthermore, Libra could lead to other BigTech responses, which could then be the basis of a range of BigTech payment systems and ecosystems (eg WhatsAppPay or Amazonbucks) rather than Libra cryptocurrency, potentially extending their global reach and influence. In addition to these, it is spurring efforts from the traditional financial industry, such as Utility Settlement Coin from FNALITY, a consortium of major financial institutions with linkages to existing central bank payment systems.^149^

At the same time, Libra is the first real rethinking of global monetary arrangements since the end of the link between the US dollar and gold in the early 1970s and the beginning of the era of floating fiat currencies. Looking forward, a tailormade international treaty-based arrangement^150^ built on a global stablecoin could offer the advantages of Libra without many of the potentially negative aspects. Highlighting how the world has changed since the announcement of Libra, Mark Carney, Governor of the Bank of England, made exactly this point in August 2019 at the high-profile annual Jackson Hole Symposium.^151^ Only time will tell whether the world is ready for a truly global stablecoin.

## Footnotes

1 See Huw Jones, ‘Facebook’s Libra Coin Closely Watched by Authorities: FSB’ (*Reuters*, 25 June 2019) <www.reuters.com/article/us-g20-fsb/facebooks-libra-coin-closely-watched-by-authorities-fsb-idUSKCN1TQ1Z5> accessed 18 August 2019.

2 See US Federal Reserve, ‘Transcript of Chair Powell’s Press Conference, June 19, 2019’ (19 June 2019) <www.federalreserve.gov/mediacenter/files/FOMCpresconf20190619.pdf> accessed 18 August 2019.

3 See Mark Carney, ‘Enable, Empower, Ensure: A New Finance for the New Economy’ (Bank of England, 20 June 2019) <www.bankofengland.co.uk/-/media/boe/files/speech/2019/enable-empower-ensure-a-new-finance-for-the-new-economy-speech-by-mark-carney> accessed 18 August 2019.

4 See Georg Meck, ‘Facebook Will Become a Major Government Creditor’ (Bundesbank, 23 June 2019) <www.bundesbank.de/en/press/interviews/-facebook-will-become-a-major-government-creditor-800042> accessed 18 August 2019.

5 See Inti Landauro, ‘France Creates G7 Cryptocurrency Task Force as Facebook’s Libra Unsettles Governments’ (*Reuters*, 21 June 2019) <www.reuters.com/article/us-facebook-crypto-france/france-creating-g7-cryptocurrency-taskforce-says-central-banker-idUSKCN1TM0SO> accessed 18 August 2019.

6 See Caroline Binham, Chris Giles and David Keohane, ‘Facebook’s Libra Currency Draws Instant Response from Regulators—G7 Countries Establish Group to Examine Risk to Financial System from “Stable Coins”’ *Financial Times* (London, 18 June 2019) <www.ft.com/content/5535fb3a-91ea-11e9-b7ea-60e35ef678d2> accessed 18 August 2019. The G7 Working Group published an initial report in October 2019, see BIS/CPMI, G7 Working Group on Stablecoins, ‘Investigating the Impact of Global Stablecoins’ (October 2019) 19 <www.bis.org/cpmi/publ/d187.pdf> accessed 11 November 2019.

7 See US House of Representatives Committee on Financial Services, Letter to Mark Zuckerberg, CEO of Facebook, Sheryl Sandberg, COO of Facebook, and David Marcus (US Government, 2 July 2019) <https://financialservices.house.gov/uploadedfiles/07.02.2019_-_fb_ltr.pdf> accessed 18 August 2019.

8 See US House of Representatives, HR 4813: Keep Big Tech Out Of Finance Act <www.congress.gov/bill/116th-congress/house-bill/4813?s=1&r=100> accessed 11 November 2019.

9 See US House of Representatives Committee on Financial Services press release, ‘Waters to Zuckerberg: You Have Opened Up a Discussion about Whether Facebook Should Be Broken Up’ (US Government, 23 October 2019) <https://financialservices.house.gov/news/documentsingle.aspx?DocumentID=404587> accessed 11 November 2019.

10 See Financial Stability Board (FSB), ‘FSB Sets Out Work to Consider Regulatory Issues of Stablecoins’ (18 October 2019) <www.fsb.org/2019/10/fsb-sets-out-work-to-consider-regulatory-issues-of-stablecoins/> accessed 26 November 2019.

11 FSB, ‘Addressing the Regulatory, Supervisory and Oversight Challenges Raised by “Global Stablecoin” Arrangements’ (14 April 2020) <www.fsb.org/wp-content/uploads/P140420-1.pdf> accessed 1 June 2020.

12 See Mehreen Khan, Sam Fleming and Caroline Binham, ‘Central Banks to Grill Facebook over Libra—Regulators Will Question Representatives of Digital Currency over Threat to Financial Stability’ *Financial Times* (London, 15 September 2019) <www.ft.com/content/11bfda8c-d6fb-11e9-8f9b-77216ebe1f17> accessed 11 November 2019.

13 See Bank of England, Financial Policy Summary and Record—October 2019, ‘Ensuring that Payment Systems Support Financial Stability’ (8 October 2019) <www.bankofengland.co.uk/financial-policy-summary-and-record/2019/october-2019> accessed 11 November 2019.

14 See Facebook, ‘Stats’ (31 March 2019) <https://newsroom.fb.com/company-info/> accessed 18 August 2019.

15 Libra Association, ‘Libra White Paper 2.0’ <https://libra.org/en-US/white-paper/> accessed 1 June 2020.

16 Focusing on legal and governance issues only, see Catherine Martin Christopher, ‘The Bridging Model: Exploring the Roles of Trust and Enforcement in Banking, Bitcoin, and the Blockchain’ (2016) 17 Nevada Law Journal 139, 140–55; Primavera De Filippi, ‘Bitcoin: A Regulatory Nightmare to a Libertarian Dream’ (2014) 3 Internet Policy Review 1, 10; Joshua J Doguet, ‘The Nature of the Form: Legal and Regulatory Issues Surrounding the Bitcoin Digital Currency System’ (2012–2013) 73 LA L Rev 1119; Reuben Grinberg, ‘Bitcoin: An Innovative Alternative Digital Currency’ (2012) 4 Hastings Science & Technology Law Journal 159, 171; Nikolei M Kaplanov, ‘NerdyMoney: Bitcoin, the Private Digital Currency, and the Case against Its Regulation’ (2012–2013) 25 Loyola Consumer Law Review 111.

17 See FA Mann, *The Legal Aspect of Money* (5th edn, Clarendon Press 1992).

18 On Libra’s regulatory qualification, see s 4 below.

19 See Christian Catalani and others, ‘The Libra Reserve’ (Libra) <https://libra.org/en-US/about-currency-reserve/?noredirect=en-US#the_reserve> at 2 accessed 18 August 2019.

20 See Ezra Klein, ‘Mark Zuckerberg on Facebook’s Hardest Year, and What Comes Next’ (*The Ezra Klein Show*, 2 April 2017) <www.vox.com/ezra-klein-show-podcast> accessed 18 August 2019.

21 See Dirk A Zetzsche and others, ‘From FinTech to TechFin: The Regulatory Challenges of Data-Driven Finance’ (2018) 14 New York University Journal of Law & Business 393.

22 See Libra Association, ‘An Introduction to Libra’ <https://libra.org/en-US/wp-content/uploads/sites/23/2019/06/LibraWhitePaper_en_US-1.pdf> accessed 18 August 2019 (hereinafter Libra 1.0 White Paper).

23 See Libra Association, ‘The Libra Association’ <https://libra.org/en-US/wp-content/uploads/sites/23/2019/06/TheLibraAssociation_en_US-1.pdf> accessed 16 August 2019; Catalani and others (n 19) 2; Zachary Amsden and others, *The Libra Blockchain* (Libra) <https://developers.libra.org/docs/assets/papers/the-libra-blockchain.pdf> accessed 18 August 2019; Libra Association, ‘Security and Privacy on the Libra Network’ <https://libra.org/en-US/wp-content/uploads/sites/23/2019/06/SecurityandPrivacyontheLibraNetwork_en_US.pdf> accessed 18 August 2019.

24 See Catalani and others (n 19) 2.

25 See Libra Association, ‘The Libra Association’ (n 23) 8.

26 See Ignacio Mas and Daniel Radcliffe, ‘Mobile Payments Go Viral: M-PESA in Kenya’ (2011) 32 Journal of Financial Transformation 169.

27 See Meeting of the Libra Association, SEC and Telegram, CME and Nasdaq (17 October 2019) <www.lh-crypto.com/crypto-news/libra-sec-and-cme.html>.

28 See Libra 1.0 White Paper (n 22) 4.

29 See nn 1–12.

30 See Commercial Registry Office Geneva, ¶HR02-1004742062.

31 ibid 8.

32 ibid 1.

33 ibid 4.

34 ibid.

35 See Libra Association, ‘The Libra Association’ (n 23) 4.

36 Own figure, based on Libra Association, ‘The Libra Association’ (n 23) 4–9. We have not included the Social Impact Advisory Board in our figure since we believe it entails social whitewashing of an otherwise financial enterprise.

37 Derived from FSB, ‘Regulation, Supervision and Oversight of Global Stablecoin Arrangements: Final Report and High-Level Recommendations’ (13 October 2020).

38 A Gevurtz, ‘Why Delaware LLCs’ (2012) 91 Or L Rev 57.

39 Arthur R Pinto, ‘The European Union’s Shareholder Voting Rights Directive from an American Perspective: Some Comparisons and Observations’ (2009) 32 Fordham Int’l LJ 587.

40 See n 30.

41 See Libra 1.0 White Paper (n 22) 4.

42 See Libra Association, ‘The Libra Association’ (n 23).

43 ibid.

44 See Lesley Fair, ‘FTC’s $5 billion Facebook Settlement: Record-Breaking and History-Making’ (*FTC Business Blog*, 24 July 2019) <https://www.ftc.gov/news-events/blogs/business-blog/2019/07/ftcs-5-billion-facebook-settlement-record-breaking-history> (detailing Facebook’s history of data protection violations and related proceedings with the FTC, including a record fine handed down in a settlement in July 2019) accessed 11 November 2019. In August 2019, Ireland’s Data Protection Commission brought 11 cases against Facebook, alleging violations of the General Data Protection Regulation in relation to Facebook’s Twitter and WhatsApp services; it is speculated that this proceeding could result in a two-digit billion euro fine, the largest for data protection violations so far; see Elizabeth Schulz, ‘Twitter and Facebook Could Be Facing Billions in Fines after Ireland Investigations’ (7 October 2019) <www.cnbc.com/2019/10/07/facebook-twitter-investigations-in-ireland-reach-conclusion.html> accessed 11 November 2019.

45 See Libra Association, ‘Security and Privacy’ (n 23) 2.

46 Christopher Babiarz, ‘Encryption Friction’ (2017) 10 Albany Government Law Review 351.

47 See Libra 1.0 White Paper (n 22) 4.

48 See Libra Association, ‘Security and Privacy’ (n 23) 4.

49 See Libra 1.0 White Paper (n 22) 4.

50 See Dirk Zetzsche, Ross Buckley and Douglas Arner, ‘The Distributed Liability of Distributed Ledgers’ [2018] U Ill L Rev 1361, 1381.

51 Prominent examples of hard forks include the cryptocurrency Ethereum, formerly the second largest cryptocurrency behind Bitcoin. From May 2017 to October 2019, this cryptocurrency experienced six hard forks, for various technical reasons (upgrades) and criminal activity. In turn, it lost its position as the second most important cryptocurrency. See ‘History of Ethereum Hard Forks: Will Istanbul Support ETH Prices?’ (7 October 2019) <https://xena.exchange/blog/history-of-ethereum-hard-forks-will-istanbul-support-eth-prices/> accessed 11 November 2019.

52 See Zetzsche, Buckley and Arner (n 49).

53 See Dirk Zetzsche and others, ‘The ICO Gold Rush: It’s a Scam, It’s a Bubble, It’s a Super Challenge for Regulators’ (2019) 60 Harv Int’l LJ 268, 288–9, 304–5.

54 On US states’ corporate law, see Jonathan Macey and Joshua Mitts, ‘ Cornell L Rev 99, 113–34 (arguing that courts are reluctant to hold an active shareholder liable for actions that are legally the responsibility of the corporation, even if the corporation has a single shareholder, while stating that courts will often do so if the corporation was markedly noncompliant with corporate formalities, to prevent fraud or to achieve equity in certain cases of undercapitalization). On English company law, see the 2013 UK Supreme Court case *Prest v Petrodel Resources Ltd* [2013] UKSC 34, [2013] 2 AC 415, ¶35 (Lord Sumption concluding that ‘there is a limited principle of English law which applies when a person is under an existing legal obligation or liability or subject to an existing legal restriction which he deliberately evades or whose enforcement he deliberately frustrates by interposing a company under his control. The court may then pierce the corporate veil for the purpose, and only for the purpose, of depriving the company or its controller of the advantage that they would otherwise have obtained by the company’s separate legal personality. The principle is properly described as a limited one, because in almost every case where the test is satisfied, the facts will in practice disclose a legal relationship between the company and its controller which will make it unnecessary to pierce the corporate veil’). On French company law, see Siel Demeyere, ‘Liability of a Mother Company for Its Subsidiary in French, Belgian, and English Law’ (2015) 3 European Review of Private Law 385, 391–402 (stating that, as a general principle, each legal person is autonomous, even when legal persons form a group, yet in some limited circumstances general contract and tort law is applied in order to hold the shareholders of a company liable, in particular in cases of fraud, concealment and the creation of false appearances). The German Bundesgerichtshof has held shareholders liable for the company’s obligations in cases of extreme undercapitalisation based on § 826 BGB (a tort law provision covering fraud), and based on another tort law provision (§ 823(2) BGB) in cases of (i) commingling of assets where the transfers between a shareholder and the company cannot be traced by viewing the books and records, (ii) disregard of corporate formalities, prompting a creditor to misperceive the corporation’s identity, (iii) fraud and (iv) misrepresentation, the latter from the basis of the Bundesgerichtshof’s doctrine of *Existenzvernichtungshaftung* (liability for destroying a company’s existence), where shareholders are liable *vis-à-vis* the subsidiary (!) for measures resulting in, or deepening, the subsidiary’s crisis. See Bundesgerichtshof, judgement of 16 July 2007, II ZR 3/04, BGHZ 173, 246—*Trihotel*. See also Christopher W Peterson, ‘Piercing the Corporate Veil by Tort Creditors’ (2017) 13 Journal of Business & Technology Law 63.

55 See Libra 1.0 White Paper (n 22) 1.

56 ibid.

57 See Douglas Arner, Ross Buckley and Dirk Zetzsche, ‘FinTech for Financial Inclusion—A Framework for Digital Financial Transformation’ (2018) <https://papers.ssrn.com/sol3/papers.cfm?abstract_id=3245287> accessed 18 August 2019.

58 See Libra 1.0 White Paper (n 22) 1.

59 See Douglas Arner and others, ‘Sustainability, FinTech and Financial Inclusion’ (2020) 21 European Business Organization Law Review 7.

60 See Elizabeth Loppatto, ‘Facebook’s Libra Probably Won’t Help People without Bank Accounts’ (*The Verge*, 27 June 2019) <www.theverge.com/2019/6/27/18760384/facebook-libra-currency-cryptocurrency-money-transfer-bank-problems-india-china> accessed 18 August 2019.

61 ibid.

62 Patrick Jenkins, ‘Why Cryptocurrencies Cannot Fix Financial Exclusion’ *Financial Times* (London, 26 June 2019) <www.ft.com/content/34ba5a70-9732-11e9-8cfb-30c211dcd229> accessed 18 August 2019.

63 See Libra 1.0 White Paper (n 22) 3.

64 See Evan Gibson, Federico Lupo Pasini and Ross P Buckley, ‘Regulating Digital Financial Services Agents in Developing Countries to Promote Financial Inclusion’ [2015] Sing JLS 26.

65 Alan Seng, ‘A World without Cash: The Future of Money’ (*Medium*, 12 February 2019) <https://medium.com/coinmonks/a-world-without-cash-the-future-of-money-ca12b31ae9bf> accessed 18 August 2019.

66 Andrew Liu, ‘An Analysis of the PBOC’s New Mobile Payment Regulation’ (2019) 39 CATO J 87.

67 Janine Aron and John Muellbauer, ‘The Economics of Mobile Money: Harnessing the Transformative Power of Technology to Benefit the Global Poor’ *European Business Review* (10 May 2019) <www.europeanbusinessreview.eu/page.asp?pid=3025>.

68 See World Bank, ‘Record High Remittances Sent Globally in 2018’ (18 April 2018) <www.worldbank.org/en/news/press-release/2019/04/08/record-high-remittances-sent-globally-in-2018> accessed 18 August 2019.

69 See World Bank, ‘Accelerated Remittances Growth to Low- and Middle-Income Countries in 2018’ (8 December 2018) <www.worldbank.org/en/news/press-release/2018/12/08/accelerated-remittances-growth-to-low-and-middle-income-countries-in-2018> accessed 18 August 2019.

70 See OECD, ‘Development Aid Drops in 2018, Especially to Neediest Countries (10 April 2019) <www.oecd.org/newsroom/development-aid-drops-in-2018-especially-to-neediest-countries.htm> accessed 18 August 2019.

71 See World Bank Group and Knomad, ‘Migration and Remittances: Recent Developments and Outlook’ (Knomad, April 2019) <www.knomad.org/sites/default/files/2019-04/Migrationanddevelopmentbrief31.pdf> accessed 18 August 2019.

72 Simon Kemp, ‘India Overtakes the USA to Become Facebook’s #1 Country’ (*The Next Web*, 13 July 2017) <https://thenextweb.com/contributors/2017/07/13/india-overtakes-usa-become-facebooks-top-country/>.

73 Henry O’Brien, ‘Uber: Payments & Profitability’ (*Medium*, 20 June 2019) <https://medium.com/swlh/uber-payments-profitability-dc171ef10b80> accessed 18 August 2019.

74 See Libra 1.0 White Paper (n 22) 3.

75 While Libra had announced in September 2019 that the basket would include 50% USD, 18% EUR, 14% yen, 11% BPD and 7% Sg-$, Libra’s CEO David Marcus announced in October, under pressure of regulators, that Libra was considering issuing a number of individual coins linked to national fiat currencies. While the basket idea necessitates global uniformity regarding the regulatory requirements as much as the risk management, if the basket idea was given up, we see very little financial inclusion delivered by Libra at all.

76 Joe Light, ‘Facebook Sees Libra Tied to Dollar, Euro, Yen but maybe not Yuan’ (*Bloomberg*, 9 September 2019) <www.bloomberg.com/news/articles/2019-09-09/facebook-sees-libra-tied-to-dollar-euro-yen-but-maybe-not-yuan> accessed 26 November 2019.

77 For instance, this could be achieved through authorised exchanges focusing on these countries, paired with certain gates and limits.

78 See Edgar Alvarez, ‘Facebook’s Calibra Cryptocurrency Wallet Launches in 2020’ (*Endgadget*, 18 June 2019) <www.engadget.com/2019/06/18/facebook-calibra-libra-cryptocurrency-digital-wallet/> accessed 18 August 2019.

79 Steven Lewis, ‘For Banks, Customer Data Is the New King’ (*Retail Banker International*, September 2013) <www.ey.com/Publication/vwLUAssets/EY_-_The_upside_of_compliance/$FILE/EY-The-upside-of-compliance-Steven-Lewis.pdf> accessed 18 August 2019.

80 Examples includes the Bank of England, the Bank of Canada, the Federal Reserve and the European Central Bank. See Michael Kumhof and Clare Noon, ‘Central Bank Digital Currencies—Design Principles and Balance Sheet Implications’ BoE Staff WP No 725/2018; Mohammad Davoodalhosseini, ‘Central Bank Digital Currency and Monetary Policy’ BoC Staff WP 2018-36; Lael Brainard, ‘Cryptocurrencies, Digital Currencies, and Distributed Ledger Technologies: What Are We Learning?’ (15 May 2018) <www.federalreserve.gov/newsevents/speech/brainard20180515a.htm> accessed 11 November 2019; ECB Crypto-Assets Task Force, ‘Crypto-Assets: Implications for Financial Stability, Monetary Policy, and Payments and Market Infrastructures’ ECB WP No 223/May 2019 <www.ecb.europa.eu/pub/pdf/scpops/ecb.op223∼3ce14e986c.en.pdf> accessed 11 November 2019.

81 Less credible experiments include the Venezuelan petro (an oil-backed cryptocurrency designed to circumvent international trade sanctions) and the Eastern Caribbean digital dollar.

82 Chen Jia, ‘China’s Digital Currency May Be World First’ *The Telegraph* (London, 9 September 2019) <www.telegraph.co.uk/china-watch/technology/china-digital-currency/> (detailing China’s efforts to digitise money supply with trusted intermediaries).

83 See Anton Didenko and Ross P Buckley, ‘The Evolution of Currency: From Cash to Cryptos to Sovereign Digital Currencies’ (2019) 42 Fordham Int’l LJ 1041.

84 Besides the efforts in China (n 81), on the German example see Ana Alexandre, ‘Germany: CDU and CSU Union to Integrate Blockchain into Public Services’ (*cointelegraph*, 25 June 2019) <https://cointelegraph.com/news/germany-cdu-and-csu-union-to-integrate-blockchain-into-public-services> accessed 18 August 2019; Frankfurter Allgemeine Zeitung, ‘Digitaler E-Euro soll Bitcoin & Co Konkurrenz machen’ *FAZ* (Frankfurt, 25 June 2019) <www.faz.net/aktuell/finanzen/digital-bezahlen/digitaler-e-euro-soll-bitcoin-co-konkurrenz-machen-16253102.html> accessed 18 August 2019 (discussing the proposal by the German Christian-Conservative Party (CDU) to issue a central bank-linked digital euro in response to Libra, and the Deutsche Bundesbank’s immediate rejection based on concerns about monetary stability, in particular a ‘digital bank-run’).

85 See FSB (n 11).

86 See Libra 1.0 White Paper (n 22) 2.

87 See Robert Kim, ‘Analysis: Facebook’s Libra Motivates Swiss Regulatory Guidance’ (*Bloomberg*, 16 September 2019) <https://news.bloomberglaw.com/bloomberg-law-analysis/analysis-facebooks-libra-motivates-swiss-regulatory-guidance> accessed 3 June 2020.

88 See Committee on Payment and Settlement Systems/Technical Committee of the International Organization of Securities Commissions, ‘Principles for Financial Market Infrastructures (April 2012) esp 126–37 <www.bis.org/cpmi/info_pfmi.htm> accessed 3 June 2020; the principles have been specified in a number of implementing standards. For instance, the Bank of England operates under the BIS framework the CHAPS (Clearing House Automated Payment System), a sterling same-day system for the settlement of high-value wholesale payments as well as time-critical, lower-value payments, like buying or paying a deposit on a property; see ‘Governance of RTGS and CHAPS’ <www.bankofengland.co.uk/payment-and-settlement>. The international accord has been implemented in the Eurosystem by the Regulation of the European Central Bank (EU) No 795/2014 of 3 July 2014 on oversight requirements for systemically important payment systems (ECB/2014/28), as amended by Regulation (EU) 2017/2094 of the European Central Bank of 3 November 2017 [2017] OJ L299/11–21; see also the additional ECB requirements ‘Revised Oversight Framework for Retail Payment Systems’ (February 2016).

89 See Directive (EU) 2015/2366 of the European Parliament and of the Council of 25 November 2015 on payment services in the internal market, amending Directives 2002/65/EC, 2009/110/EC and 2013/36/EU and Regulation (EU) No 1093/2010, and repealing Directive 2007/64/EC [2015] OJ L337/35 (PSD2).

90 Libra intends to use the Libra Reserve for a number of purposes, including participation of Libra holders, business expansion and dividend payments to members of the Libra Association. Under payment systems regulations, the provider bears in principle all the risk from establishing and running the system, while Libra holders are prevented from benefits accrued in the guarantee fund; further, the Libra documents remain silent on the segregation and custody requirements which apply to a payment system’s guarantee funds.

91 See FINMA press release, ‘FINMA äussert sich zu ‘Stable Coins’’ (11 September 2019) <www.finma.ch/de/news/2019/09/20190911-mm-stable-coins/> accessed 3 June 2020.

92 30 countries after the UK’s departure from the EU.

93 PSD2 (n. 88) art 10(1).

94 See art 2(8) of Directive 2009/110/EC of the European Parliament and of the Council of 16 September 2009 on the taking up, pursuit and prudential supervision of the business of electronic money institutions amending Directives 2005/60/EC and 2006/48/EC and repealing Directive 2000/46/EC [2009] OJ L267/7.

95 Sharon Brown-Hruska and Trevor Wagener, ‘The Virtual Currency Regulatory Framework in Global Context’ (2018) 13 CMLJ 487, 507–8 (for a discussion of the NYDFS ‘BitLicense Regulatory Framework’); Anastasia Sotiropoulou and Dominique Guegan, ‘Bitcoin and the Challenges for Financial Regulation’ (2017) 12 CMLJ 466, 475–6 (outlining the NYDFS ‘Bitlicense’ and the Japanese virtual currency regime).

96 Sotiropoulou and Guegan (n 94) 475 (discussing the application of German and French existing licensing requirements to virtual currency providers).

97 See Dirk Zetzsche, ‘The Anatomy of European Investment Law’ in William Birdthistle and John Morley, *Research Handbook on the Regulation of Mutual Funds* (Edward Elgar 2018) 302, 335–8.

98 See Directive 2009/65/EC of the European Parliament and of the Council of 13 July 2009 on the coordination of laws, regulations and administrative provisions relating to undertakings for collective investment in transferable securities (UCITS), [2009] OJ L302/32, as amended. On the UCITS framework, the world’s most recognised set of rules for retail investment funds, see generally ibid 302, 305–9, 338–40.

99 For an argument qualifying Libra as a money market fund from the US perspective in line with the arguments outlined in this article, see Written Testimony of Chris Brummer, Professor of Law, Georgetown University Law Center before the United States House Committee on Financial Services Examining Facebook’s Proposed Cryptocurrency and Its Impact on Consumers, Investors, and the American Financial System ‘99 Problems’, 17 July 2018.

100 See Catalani and others (n 19) 2.

101 Details of the entitlement in the pool need to be determined, and will be relevant for the fund qualification of Libra.

102 See William Birdthistle, ‘*Breaking Bucks* in Money Market Funds’ [2010] Wis L Rev 1155, 1159 (highlighting the conservative investment policy of money market funds).

103 ibid 1176–8 (describing the growth and crisis of US money market funds).

104 International Organization of Securities Commissions, ‘Update to the IOSCO Peer Review of Regulation of Money Market Funds’ (*Bloomberg*, October 2019) <www.bloomberg.com/news/articles/2019-09-09/facebook-sees-libra-tied-to-dollar-euro-yen-but-maybe-not-yuan> accessed 26 November 2019.

105 See BIS/CPMI (n 6).

106 See Zetzsche and others, ‘The ICO Gold Rush’ (n 52).

107 See Brown-Hruska and Wagener (n 94) 497–508 (discussing different classification of virtual currencies taken up by various US agencies and courts).

108 See BIS/CPMI (n 6).

109 See Directive 2014/65/EU of the European Parliament and the Council of 15 May 2014 on markets in financial instruments and amending Directive 2002/92/EC and Directive 2011/61/EU (recast) [2014] OJ L173/349.

110 The ‘investment services and activities’ defined in Annex I, s A of the MiFID refers to the definition of ‘financial instruments’ in Annex I, s C of the MiFID (see n 108). The latter includes, inter alia, ‘Options, futures, swaps, forward rate agreements and any other derivative contracts relating to … currencies … which may be settled physically or in cash’. On the MiFID licensing rules, see generally Dirk Zetzsche, ‘Article 5 MiFID II’ in Matthias Lehmann and Christoph Kumpan, *European Financial Services Law—Article-by-Article Commentary* (Beck/Nomos/Hart Publishing 2019); on the concept of ‘investment firm’ under MiFID, see Matthias Lehmann, ‘Article 4 MiFID II’ in Lehmann Kumpan (ibid) ¶¶4–10.

111 The website *cointelegraph* lists for the first half of 2019 seven successful attacks on cryptoexchanges with damage exceeding US$75 million and for the whole of 2018 seven successful attacks with damage of at least US$750 million. See Joseph Young, ‘Round Up of Crypto Exchanges Hacks So Far in 2019: How Can It Be Stopped? (*cointelegraph*, 18 June 2019) <https://cointelegraph.com/news/round-up-of-crypto-exchanges-hack-so-far-in-2019-how-can-it-be-stopped> accessed 11 November 2019. For a list of the most prominent hacks, including the notorious Mt Gox hack of 2014, see Zetzsche, Buckley and Arner (n 49) 1367–9.

112 See Dennis Chu, ‘Broker-Dealers for Virtual Currency: Regulation Cryptocurrency and Exchanges’ (2019) 118 Colum L Rev 2323, 2343–6 (discussing risks of cryptoasset exchanges).

113 ibid 2352–9 (discussing regulation of cryptoasset exchanges).

114 See nn 108 and 109.

115 See art 16(8)–(11) of MiFID (n 108), as further detailed in arts 2–7 of the Commission Delegated Directive (EU) 2017/593 of 7 April 2016 with regard to safeguarding of financial instruments and funds belonging to clients, product governance obligations and the rules applicable to the provision or reception of fees, commissions or any monetary or non-monetary benefits. On the MiFID safeguarding and segregation rules, see Dirk Zetzsche, ‘Article 16 MiFID II’ in Lehmann and Kumpan (n 109) ¶¶45–63.

116 See the sanctioning rules for data protection violations as detailed in art 83 et seq of the European General Data Protection Regulation (EU) 2016/679 (GDPR), allowing for penalties of up to 4% of a firm’s group-wide turnover.

117 On cyberrisk, see Ross Buckley and others, ‘The Dark Side of Digital Financial Transformation: The New Risks of FinTech and the Rise of TechRisk’ [2020] Sing JLS (forthcoming), available as EBI Working Paper <www.ssrn.com/abstract=3478640>.

118 See Libra 1.0 White Paper (n22) 3.

119 See BIS/CPMI (n 6).

120 As we know from the fund domain, synthetic exposures create a significant degree of counterparty risk, with the respective Libra baskets functioning as counterparties. Hence, risk management will be crucial.

121 See *Handelsblatt* (Düsseldorf, 28 June 2019), citing Philip Sander, head of the Blockchain Centre in Frankfurt.

122 Buckley and others (n 116).

123 See FSB, ‘Addressing SIFIs’ <www.fsb.org/work-of-the-fsb/policy-development/addressing-sifis/> accessed 11 November 2019 (stating that ‘Systemically important financial institutions (SIFIs) are financial institutions whose distress or disorderly failure, because of their size, complexity and systemic interconnectedness, would cause significant disruption to the wider financial system and economic activity. At the Pittsburgh Summit in 2009, G20 Leaders called on the FSB to propose possible measures to address too-big-to-fail (TBTF) problems associated with SIFIs … The objective of the SIFI Framework is to address the systemic risks and the associated moral hazard problem for institutions that are seen by markets as TBTF. It does so by reducing the probability of SIFIs failing through implementation of a multipronged and integrated set of policy measures’).

124 The FSB, in consultation with the Basel Committee on Banking Supervision (BCBS) and national authorities, has identified global systemically important banks (G-SIBs) since 2011, and, in consultation with the International Association of Insurance Supervisors (IAIS) and national authorities, began identifying global systemically important insurers (G-SIIs) in 2013. See FSB, ‘Global Systemically Important Financial Institutions (G-SIFIs)’ <www.fsb.org/work-of-the-fsb/policy-development/addressing-sifis/global-systemically-important-financial-institutions-g-sifis/> accessed 11 November 2019.

125 See Douglas W Arner, Janos Barberis and Ross P Buckley, ‘The Evolution of FinTech: A New Post-Crisis Paradigm?’ (2016) 47 Georgetown Journal of International Law 1271.

126 See Zetzsche and others, ‘From FinTech to TechFin’ (n 21).

127 See Dirk A Zetzsche and others, ‘The Future of Data-Driven Finance and RegTech: Lessons from EU Big Bang II’ EBI Working Paper 2019/35 <https://ssrn.com/abstract=3359399> accessed 18 August 2019.

128 Karen Yeung, ‘Regulation by Blockchain—the Emerging Battle for Supremacy between the Code of Law and Code as Law’ (2019) 82 MLR 207, 217 (discussing US Financial Crimes Enforcement Network 2013 guidelines and successful suit against Ripple Labs Inc).

129 See Libra 1.0 White Paper (n 22) 9.

130 See Douglas Arner and others, ‘The Identity Challenge in Finance: From Analogue Identity to Digitized Identification to Digital KYC Utilities’ (2019) 20 EBOR 55.

131 See Libra 1.0 White Paper (n 22) 9.

132 See Arner and others, ‘The Identity Challenge’ (n 129).

133 See n 43.

134 Ross P Buckley and Sarala M Fitzgerald, ‘An Assessment of Malaysia’s Response to the IMF during the Asian Economic Crisis’ [2004] Sing JLS 96.

135 See Libra Association, ‘The Libra Association’ (n 23) 1.

136 See Alvarez (n 77).

137 See Lee Reiners, ‘What Congress Should Ask about Facebook’s New Cryptocurrency’ (*The FinReg*, 2 July 2019) <https://sites.duke.edu/thefinregblog/2019/07/02/what-congress-should-ask-about-facebooks-new-cryptocurrency/> accessed 18 August 2019.

138 See Zetzsche and others, ‘The ICO Gold Rush’ (n 52).

139 Members of an association are not entitled to dividends under Swiss law; we speculate that at least the founding members of the Libra Association are, or will become, shareholders of the Libra Networks s.a.r.l.

140 See Libra 1.0 White Paper (n 22) 7.

141 In line with this estimate, a recent survey among regulators asking for the regulatory approaches to stablecoins revealed that the national perspective differs from country to country, with the frameworks for e-money, money market funds and securities all scoring in the 50% range, indicating widely spread disagreement among regulators.

142 See generally Howell E Jackson, ‘Substituted Compliance: The Emergence, Challenges, and Evolution of a New Regulatory Paradigm’ (2015) 1 Journal of Financial Regulation 169; Dirk A Zetzsche, ‘Competitiveness of Financial Centers in Light of Financial and Tax Law Equivalence Requirements’ in Ross P Buckley, Emilios Avgouleas and Douglas W Arner (eds), *Reconceptualizing Global Finance and its Regulation* (CUP 2016) 390, 390–418.

143 On China, see Fred Imbert, ‘Trump Accuses China of “Currency Manipulation” as Yuan Drops to Lowest Level in More Than a Decade’ (*CNBC*, 5 August 2019) <www.cnbc.com/2019/08/05/trump-accuses-china-of-currency-manipulation-as-yuan-drops-to-new-low.html>. On Europe, see Weizhen Tan, ‘Trump Tweet Sparks Concerns That the US May Raise Tariffs on Europe’ (*CNBC*, 4 July 2019) <www.cnbc.com/2019/07/04/trump-tweet-on-china-europe-fx-manipulation-raises-eu-tariff-fears.html>.

144 See Hearing before the United States Senate Committee on Banking, Housing, and Urban Affairs, Testimony of David Marcus, Head of Calibra, Facebook (16 July 2019) <www.banking.senate.gov/imo/media/doc/Marcus%20Testimony%207-16-19.pdf> accessed 5 June 2020.

145 On the SSM, see Tobias Tröger, ‘The Single Supervisory Mechanism—Panacea or Quack Banking Regulation? Preliminary Assessment of the New Regime for the Prudential Supervision of Banks with ECB Involvement’ (2014) 15 EBOR 449.

146 Tom Warren, ‘Facebook Pay Is a New Payment System for WhatsApp, Instagram, and Facebook’ (*The Verge*, 12 November 2019) <www.theverge.com/2019/11/12/20961447/facebook-pay-whatsapp-instagram-messenger-features> accessed 26 November 2019.

147 Daniel Keyes, ‘WhatsApp Pay Is on the Verge of Launching in India’ (*Business Insider*, 2 July 2019) <www.businessinsider.com/whatsapp-pay-ready-for-india-launch-2019-7> accessed 26 November 2019.

148 Yuan Yuan and Hudson Lockett, ‘What Is China’s Digital Currency Plan?’ *Financial Times* (London, 26 November 2019) <www.ft.com/content/e3f9c3c2-0aaf-11ea-bb52-34c8d9dc6d84> accessed 26 November 2019; Alun John, ‘China’s Digital Currency Will Kick Off ‘Horse Race’: Central Bank Official’ (*Reuters*, 6 November 2019) <www.reuters.com/article/us-china-markets-digital-currency/chinas-digital-currency-will-kick-off-horse-race-central-bank-official-idUSKBN1XG0BI> accessed 26 November 2019; Naomi Xu Elegant, ‘Why China’s Digital Currency is a “Wakeup Call” for the US’ (*Fortune*, 1 November 2019) <https://fortune.com/2019/11/01/china-digital-currency-libra-wakeup-call-us/> accessed 26 November 2019.

149 See www.fnality.org/.

150 See International Monetary Fund, Articles of Agreement, art IV, s 2(c) (permitting the establishment of new general exchange arrangements).

151 Mark Carney, ‘The Growing Challenges for Monetary Policy in the Current International Monetary and Financial System’ (Jackson Hole Symposium, 23 August 2019) <www.bankofengland.co.uk/-/media/boe/files/speech/2019/the-growing-challenges-for-monetary-policy-speech-by-mark-carney.pdf> accessed 26 November 2019.

